# Design analysis and performance enhancement of a 2-element MIMO skin-implantable antenna for IoT-based health monitoring devices

**DOI:** 10.1371/journal.pone.0311753

**Published:** 2024-12-05

**Authors:** Anupma Gupta, Mohammad Aljaidi, Shonak Bansal, Rabia Emhamed Al Mamlook, Vipan Kumar, Abeer Aljohani, Saad Aljohani, Manish Kumar Singla

**Affiliations:** 1 Department of Interdisciplinary Courses in Engineering, Chitkara University Institute of Engineering and Technology, Chitkara University, Punjab, India; 2 Department of Electronics and Communication Engineering, Saveetha School of Engineering, Saveetha Institute of Medical and Technical Sciences, Thandalam, Chennai, Tamilnadu, India; 3 Department of Computer Science, Faculty of Information Technology, Zarqa University, Zarqa, Jordan; 4 Department of Electronics and Communication Engineering, University Institute of Engineering, Chandigarh University, Gharuan, Mohali, India; 5 Department of Mechanical and Industrial Engineering, University of Zawia, Zawia, Libya; 6 Department of Electronics and Communication Engineering, Sri Sai College of Engineering and Technology, Badhani, Pathankot, India; 7 Applied College Taibah University, Madinah, Saudi Arabi; 8 Electrical and Computer Engineering, Western Michigan University, Kalamazoo, Michigan, United States of America; 9 Jadara University Research Center, Jadara University, Irbid Jordan; Model Institute of Engineering and Technology, INDIA

## Abstract

Two two-element slotted patch multiple-input multiple-output (MIMO) antenna with coplanar waveguide (CPW) feed is proposed for deployment in implantable medical devices. Implantable devices are compact and demand high-gain antennae with unidirectional radiation patterns. Regarding compactness, the antenna has a size of 16 × 6×0.25 mm^3^ for the operating frequency of 2.45 GHz ISM band. Miniaturized antenna geometry is attained using the slots in the radiator and with the coplanar waveguide feed. An element MIMO configuration is used for establishing a reliable communication link. The two radiators maintain a connected ground topology with a minimum edge-to-edge spacing of 7.7 mm. Mutual coupling of -29 dB and operating frequency spectrum of 1300 MHz is attained at the center cut off frequency. Unidirectional power pattern and high gain of 2.3 dB is achieved. The proposed MIMO structure is suitable for mitigating multipath fading effect and realizing higher spectral efficiency in short range communication. The in-tissue measurement results for the scattering parameters and radiation characteristics are done by placing the antenna in a pork loin. Specific absorption rate (SAR) analysis is performed to confirm the user’s safety from nearfield EM waves. Antenna has safe SAR value of 0.798 W/kg for 1-g of average tissue for the power of 1-mW. The low envelope correlation coefficient of 0.035 depicts the excellent diversity performance for MIMO operation.

## I. Introduction

In the last few years, the health industry has been revolutionized with different technologies by remote access and diagnosis of medical situations of patients. In the realm of IoT-based medical technology, the development of implantable medical devices (IMDs) is a leading-edge area that continually sparks new enthusiasm within the industry [1–4]. It involves a reliable and robust wireless communication link to transfer real-time time parameters including, heartbeats, electroencephalograms (EEGs) [5, 6], electrocardiograms (ECGs), blood sugar level, temperature, pH values. In addition to this the wireless implantable neurostimulator, a novel digital therapeutic instrument, holds significant clinical value for conditions such as epilepsy, Parkinson’s and many related ailments [7–12]. An implantable antenna is a crucial part of the IMDs with several challenges including physical space limitation on the chip, wide bandwidth, robustness to operate in different biological surrounding tissues, and assurance of patient safety from EM waves.

Different approaches adopted by researchers to deal with these challenges [13–27]. In [13, 14] multiband resonance is achieved to support the different communication standards. Circular polarization characteristics are integrated to mitigate the orientation mismatch issues in [19, 20, 25]. Wideband operation is achieved by using PIFA structures [15], parasitic patches [18], multiple slots [23], complex spiral-shaped feedlines [24], and adding ladder slots and capacitors in the ground plane [26]. Wide bandwidth resonance avoids the variation in operating frequency due to multilayered tissue effect. The physical size of the antenna is reduced to ease the integration of the antenna by etching meandered slots in the radiator [17], inserting shorting pins with T-shaped slots [21], defected ground with spiral-shaped radiator [22], and asymmetrical slotted ground [27] structure. The efficiency of the implant antenna is improved to lessen the absorption of EM waves [16]. However, recent literature relies on the single radiator based configurations, which suffer from multipath reflections fading effect and signal distortion, especially during indoor propagation [28, 29]. On the contrary, the deployment of MIMO configuration grants the potential to improve spectral efficiency and channel capacity, supports high data rate without using much power and bandwidth [28] in medical applications.

Some of the latest research for implantable MIMO antennae is presented in [30–44]. The primary challenge in MIMO antenna design for IMDs lies in achieving miniaturization within constrained area for enhancing isolation between radiators [21]. In [30], a miniaturized structure employing a 4-port MIMO in the ISM band is proposed, Miniaturization is realized through a shared radiator structure, and mutual coupling is reduced by via-embedded electromagnetic band gaps (EBGs). Another design [31] introduces a MIMO antenna with meandered patch to achieve miniaturization and reduce coupling through the use of substrates with higher permittivity, and defected ground. Characteristics Mode Analysis Theory is used to excite the decoupled mode in the MIMO radiators [32, 33]. In [34] circular slotted patch is used for size reduction and a high impedance surface is integrated for mutual coupling reduction. An orthogonal radiator arrangement is proposed in [35] along with a central metallic via for port isolation. Cube cube-shaped MIMO antenna with circular-slotted radiators on the sides of cubes is proposed in [36]. A semicircular radiator with shorting pins and a slotted ground structure is proposed in [37, 38]. For cylindrical-shaped ingestible MIMO antenna configuration, orthogonal loop radiators [39], and a cylindrical-shaped metallic bar [40], cross-coupled slot [41] is used to mitigate the coupling effect in radiators. Thus, various methods like meandered patch, adding of vias, slotting, substrates with high dielectric constant, and cubic geometry have been adopted to achieve smaller sizes and efficient coupling reduction. These decoupling techniques enhance the complexity of structures intricate the fabrication process and challenge the installation of an antenna within the implanted device. Therefore, the primary focus remains on achieving compactness of MIMO antennas while improving port isolation within the limited space. In addition to this, existing in-body MIMO antennas possesses narrow-band spectrum. To ensure the robustness of communication links, the wide-band operation is advantageous. Therefore, development of in-body MIMO antenna with a wide operating spectrum, small and simple geometry, and excellent diversity performance is highly required.

In this paper, two port MIMO structure for deep tissue implant is suggested. It shows robust in-body operation, wider bandwidth, and high port isolation, and ensures tissue safety. It operates at a 2.45 GHz ISM band with an operating bandwidth of 1.3 GHz (from 1.6 GHz to 2.9 GHz). MIMO structure is designed to mitigate channel interference in the dense short-range communication channel. Slotted patch geometry is used to tune the antenna and a T-shaped decoupling network with the connected ground plane is employed for mutual coupling reduction. Decoupling networks offset the radiations from one patch to another. It restricts the flow of electromagnetic waves toward the adjacent port, thereby enhancing the isolation. Over the operating bandwidth of less than -20 dB mutual coupling is obtained for the extremely closely placed radiators. The antenna has shown excellent radiation characteristics with a unidirectional radiation pattern and gain of 2.31 dB. The proposed MIMO system is simulated in the different biological tissues and measurements are performed in a pork loin. The antenna has stable frequency and unidirectional radiation behavior with a low SAR value.

## II. Tissue-phantom models for simulation set-up

**Fig 1:**
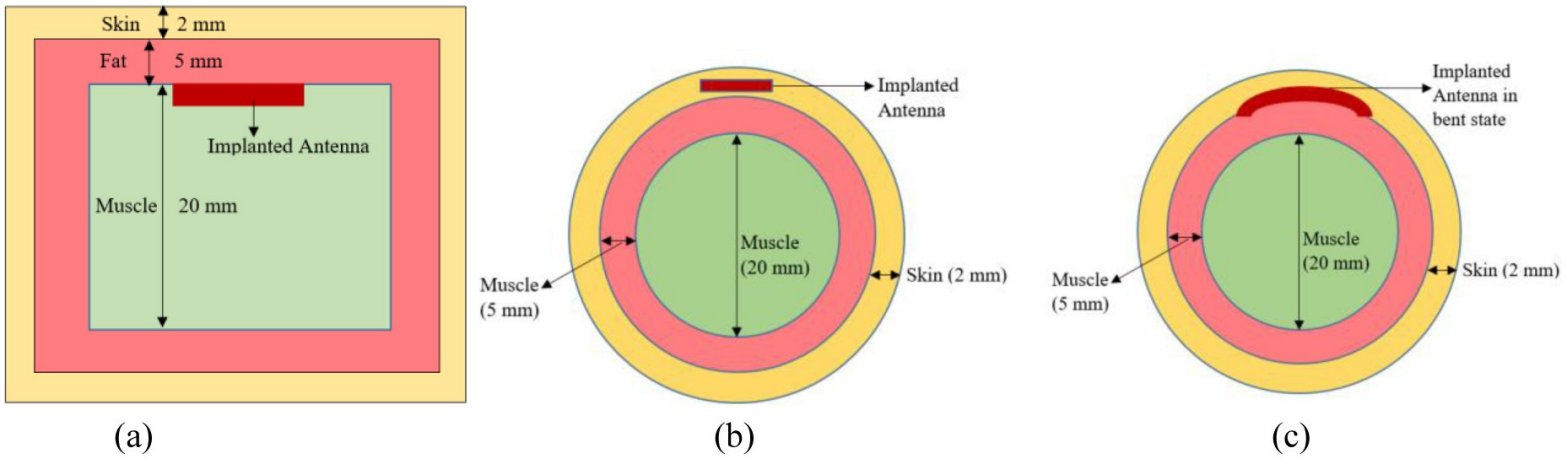
A simulation model of a 3-layered tissue phantom antenna is implanted in (a) rectangular muscle tissue (b) circular skin tissue and (c) under bending conditions.

The design process considers the implant environment within the body. A numerical model is created to represent the layers of tissue the antenna will be placed in. As studied in literature, each body tissue layer has a specific thickness, Skin thickness varies form 1.5 mm to 3 mm. Fat thickness varies from 2mm to 5mm and muscle thickness ranges from 8 mm to 100 mm. In this research, three layered tissue model is designed with the average tissue thickness Muscle tissue (innermost layer, 20mm thick), fat tissue (middle layer, 5mm thick), and Skin tissue (top layer, 2mm thick). Implant devices are implanted between different layers according to the application. Here, antenna is analyzed to be implanted in different layers: skin layer at 2 mm in circular phantom and 5 mm in muscle layer of rectangular tissue. A brief analysis of the different implant depth used by the researchers in mentioned in Table 2. This model includes; Muscle tissue (innermost layer, 20mm thick), fat tissue (middle layer, 5mm thick), and Skin tissue (top layer, 2mm thick). These thicknesses are average and can vary depending on factors like body type [37, 38, 42]. Electric properties of three tissue layers are: skin (εr = 38 and σ = 1.46 s/m), fat (εr = 5.2 and σ = 0.10 s/m) and muscle (εr = 52.7 and σ = 1.8 s/m) [1]. For the simulation, the tissue phantom is modeled as a rectangular block with dimensions of 60mm x 60mm as shown in Fig 1(a). The antenna is positioned within the muscle layer. Implant depth is studied from the existing literature and briefed in Table 1. Realistically, implantable devices can go in various tissues with different shapes. To assess this, the antenna is placed in the skin layer of a cylindrical phantom with a diameter of 27mm (Fig 1b), simulating a different implant location. In addition to this, implantable antennas are needed to bend according to the shape of the organ. Thus, antenna behavior is studied for the circular shaped organs and deformation effect. The antenna is bent across the circular phantom and analyzed for the reliability of the antenna for real-time applications. For initial design and analysis, the rectangular phantom of Fig 1(a) is considered.

**Table 1:** Implant depth analysis from the literature.

Reference	Implant Tissue layer	Implant Depth
[45]	Skin	3 mm
[46]	Skin, muscle	1.6 mm, 7 mm
[47]	Brain implant (dura layer)	12mm
[22]	skin	2 mm
[31]	muscle	75 mm
This Work	Skin, muscle	2 mm, 7mm

**Fig 2:**
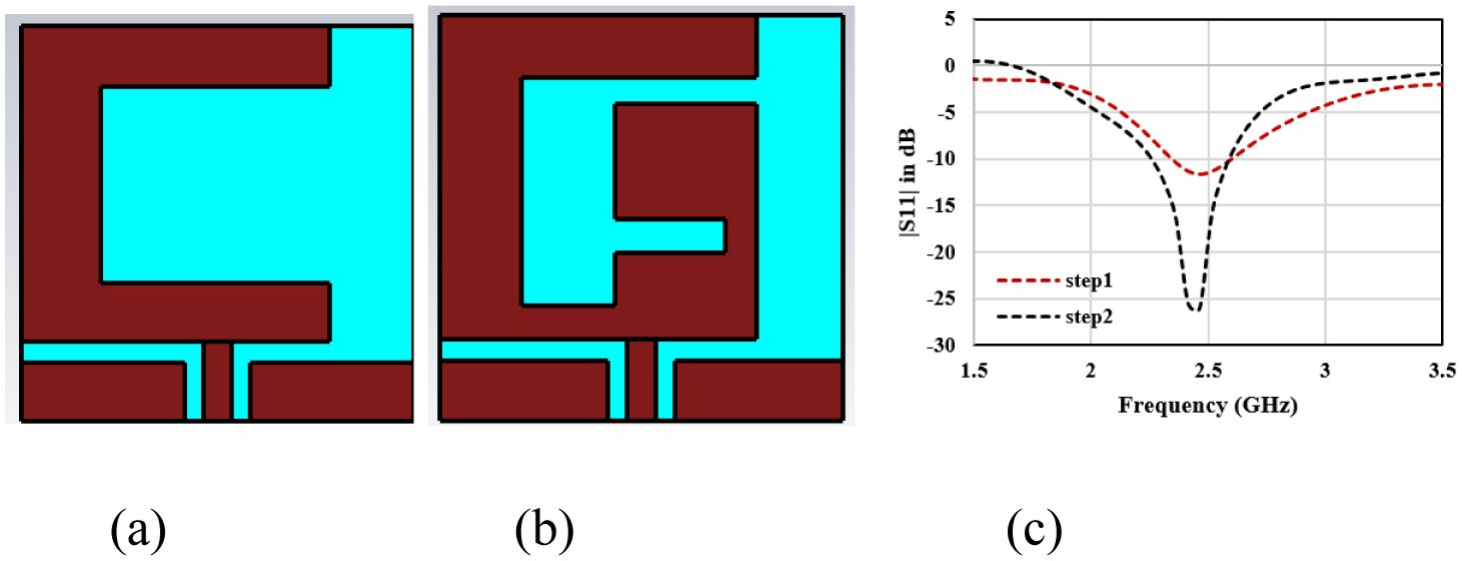
Single element design (a) step1 design (2) step2 design (c) Reflection Coefficicent |S11| for single eleement design steps.

**Fig 3:**
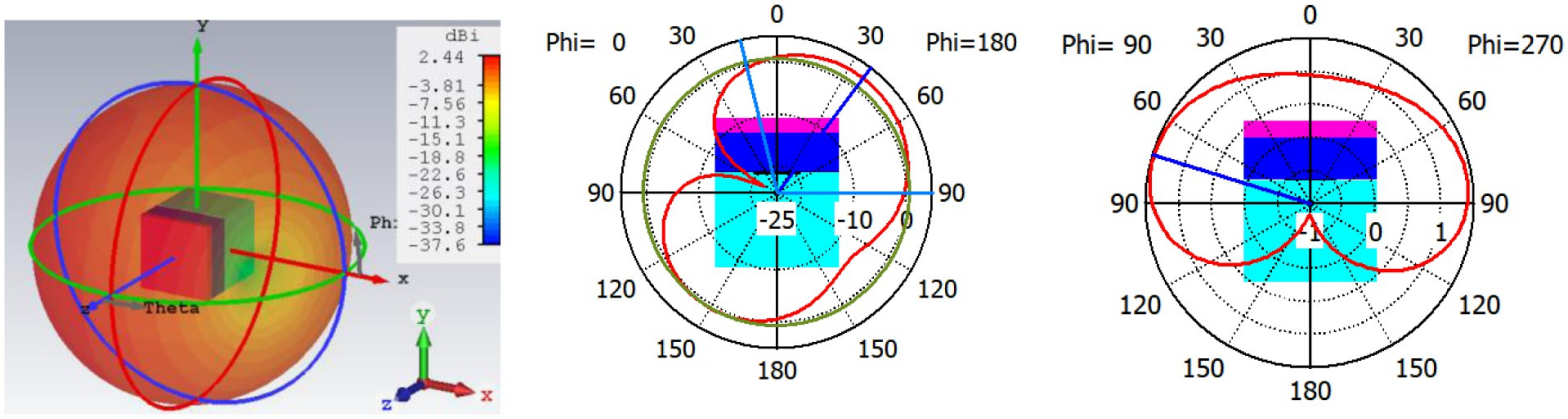
(a) 3-D radiation plot (b) E-plane (c) H-plane radiation characteristics of single element structure.

A single radiator in-body antenna is initially designed and simulated. A muscle tissue implanted coplanar waveguide fed patch antenna is designed on dielectric-material Rogers 5880 with a thickness of 0.125mm, ε_r_ “(relative permittivity) value of 2.2 and δ (loss tan) value of 0.0009.” The planar size of the single-element structure is 8 mm×6 mm which is equivalent to 0.11λo × 0.08λo, where λo represents the free space wavelength at 2.45 GHz. A G-shaped radiator is used to tune the antenna at 2.45 GHz. Fig 2(a) and 2(b) shows the design of the single radiator structure. It is evaluated in two steps. In step1, C-shaped radiator is designed and optimized to attain the frequency response at 2.45 GHz. Reflection coefficient plot in Fig 2(c) shows that antenna has reflection coefficient value of 11 dB is achieved at 2.45 GHz. Implantable device antenna requires better impedance matching with higher value of reflection coefficient as complex body tissue structure may degrades the impedance matching and resonance frequency when deployed in different person and organs. Therefore, antenna is modified in step2 by adding an inverted C-shaped radiator in the antenna. It leads to shifts the reflection coefficient to 25 dB with a bandwidth of 600 MHz form 2.0 GHz to 2.6 GHz. Radiation characteristics of the single radiator antenna is shown in Fig 3. Antenna has gain of 2.44 dBi at 2.45 GHz. In E-plane, bidirectional radiation pattern and in H-plane unidirectional radiation pattern is observed. In both the planes, maximum radiations are oriented towards the normal to the body surface which is the required characteristics of in-body antenna.

**Fig 4:**
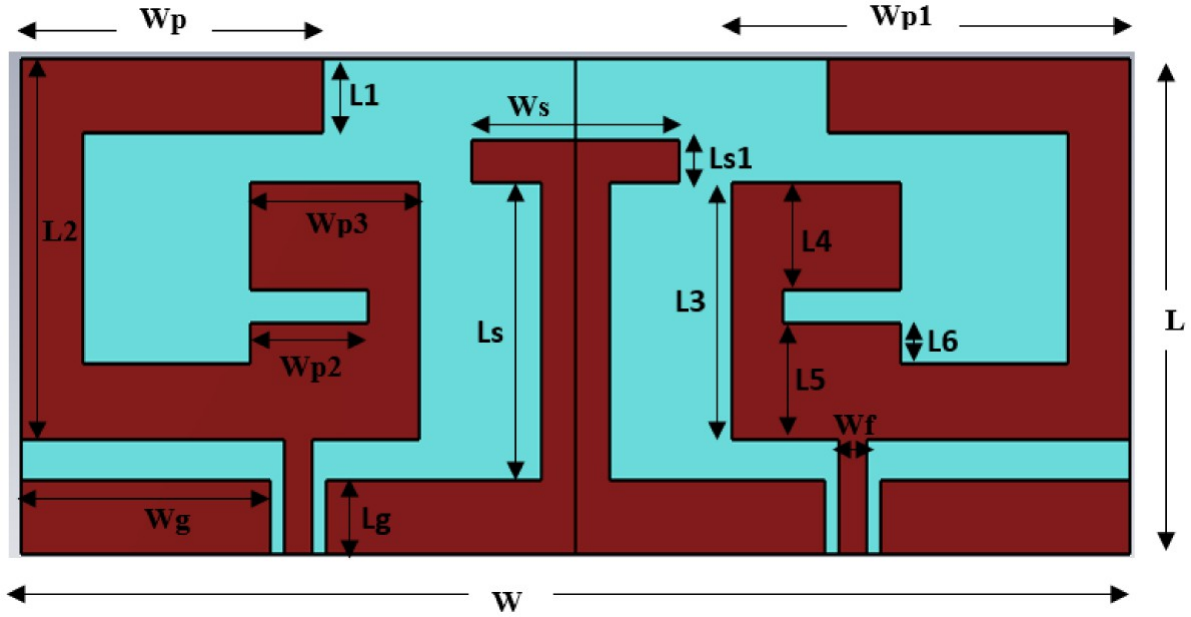
Geometrical view of the 2-element MIMO antenna.

Further, single radiator antenna is modified and optimized to be used in MIMO configuration. The topology of the 2-element MIMO structure is represented in Fig 4. Identical radiators are placed in the complementary symmetry configuration. T-shaped stub is added with the ground plane in the middle of two radiating patches which contributes to reducing the mutual coupling between the radiators. The conducting part of the antenna is isolated from body tissue layers by adding a layer of the dielectric material RO3010 above the conducting part with a thickness of 0.125mm, “ε_r_ (relative permittivity) value of 10.2 and δ (loss tan) value of 0.0022.” Table 2 lists the geometrical parametric values of the 2-element MIMO antenna.

**Table 2:** Geometrical parameters value of 2-element MIMO antenna.

parameter	Wp	Wp1	W	Wp2	Wp3	Wg
Value (mm)	4.15	5.55	16	2	3.55	3.6
parameter	L	L1	L2	L3	L4	L5
Value (mm)	6	0.9	4.6	3.1	2.3	2.2
parameter	Ls	Lg	Ls1	Ws	Wf	L6
Value (mm)	6.1	0.9	0.5	3	0.4	0.5

**Fig 5:**
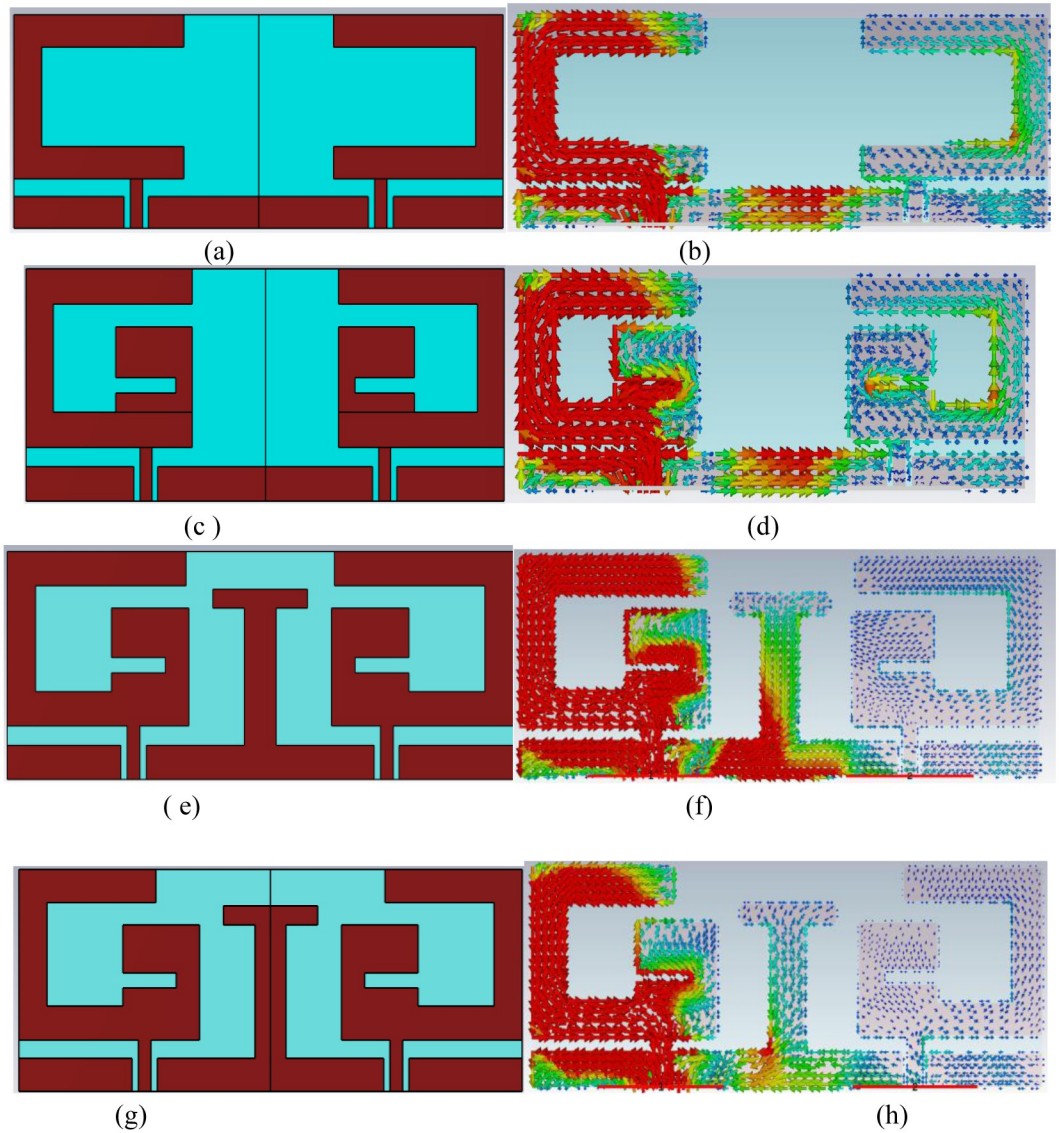
Stepwise design topologies and surface current distribution (a and b) design1 (c and d) design2 (e and f) Design3 (g and h) Design4(Proposed structure).

**Fig 6:**
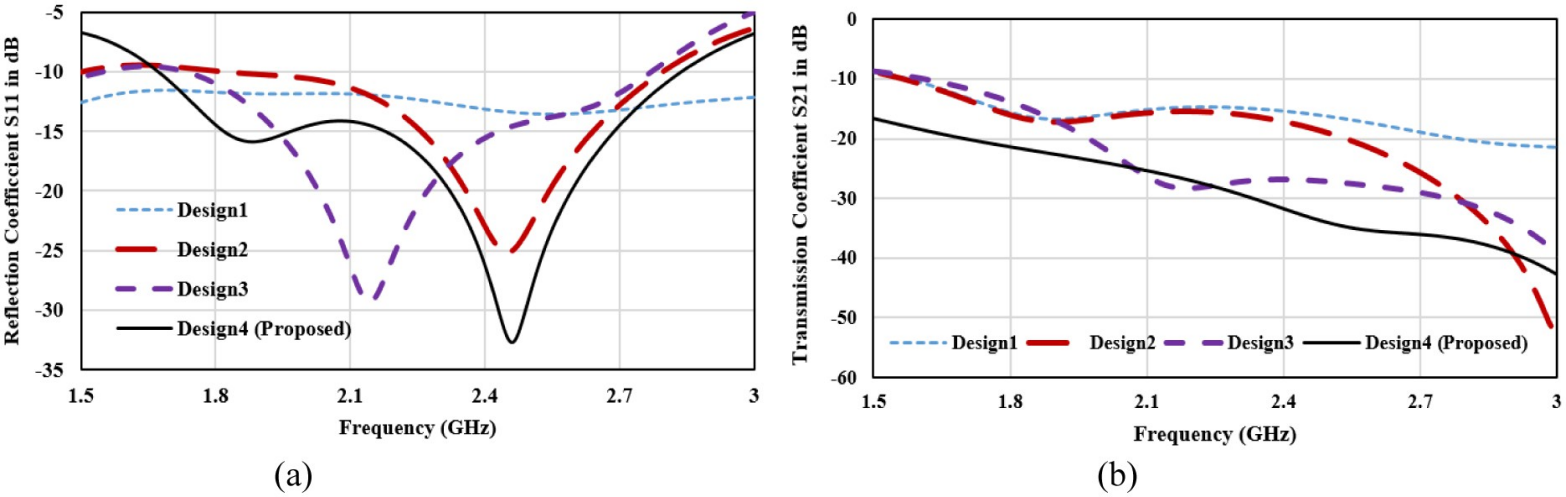
(a) Reflection coefficient plot for step wise antenna design (b) Transmission coefficient plot for step wise antenna design.

**Fig 7:**
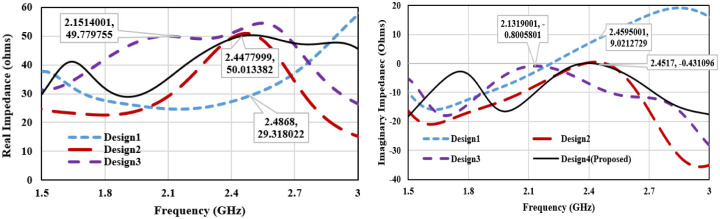
(a) Real-impedance plot for step-wise antenna design (b) Imaginary-impedance plot for step-wise antenna design.

The MIMO antenna-design process is described through 4-stepwise designs and their surface current distribution which is shown in Fig 5. The reflection coefficient and Transmission coefficient plots of 4-designs are depicted in Fig 6. In design1, two C-shaped radiators are arranged in complementary symmetry configurations. Coplanar ground for the two radiators is connected. C-shaped radiator is tuned at 2.45 GHz, as shown in Fig 4(a). Design1 S11 curve depicts that the reflection coefficient plot is below -10 dB over the wide spectrum. This is not satisfactory for in-body communications. Hence, design1 is further modified to enhance the antenna performance and get the desired bandwidth and scattering parameters. The surface current plot for design shows that a single resonance path is excited in the antenna. Therefore, the impedance characteristics of the antenna are observed, and the antenna structure is modified accordingly. Fig 7. shows the real and imaginary impedance curves for all the design topologies.

In design1, reactance is 9.02 ohms and resistance is about 29.3 Ohms at 2.45 GHz. To nullify the reactance and get the impedance of 50 ohms, an inverted C-shaped patch is added to the main C-shaped radiator. It converts the patch into a G-shape and alters the impedance. In design2, 50-ohms impedance with null reactance has been attained. Attaching the second c-shaped patch in the radiator has increased the resistance and inductive reactance and reduced the capacitive reactance. The reflection coefficient plot for Design2 in Fig 6(a) depicts that the impedance bandwidth for the designed structure is 750 MHz (2.0 to 2.75 GHz) with a reflection coefficient of -25 dB at 2.45 GHz. Transmission coefficient plot for design2 in Fig 6(b) shows that mutual coupling between the radiators is about -15dB which is not suitable for the MIMO configuration. Thus, a decoupling network is required to isolate the radiators, and antenna topology is modified further.

In Design 3, a T-shaped decoupling radiator is attached to the connected ground plane. It improves the isolation of about -28 dB over the entire operating bandwidth. T-shaped decoupling radiator is attached to the connected ground plane to enhance the isolation. Isolation stub is added at the location where maximum current is coupled from one port to another port. Here, the connected coplanar ground has the maximum intensity of coupling power. Thus, a T-shaped stub is added at the connected ground. Antenna impedance is matched by taking the dimension of T-shaped stub approximately equivalent to the resonance current path length calculated from Eq (5). Length of the T-shaped stub (L_stub_) is approximately 18.0 mm. (L_stub_ = 2(Ls)+Ws+2Ls1+2Lg). It acts as a parasitic resonator at frequency 2.45 GHz and maintains the impedance matching after adding the isolation stub.

**Fig 8:**
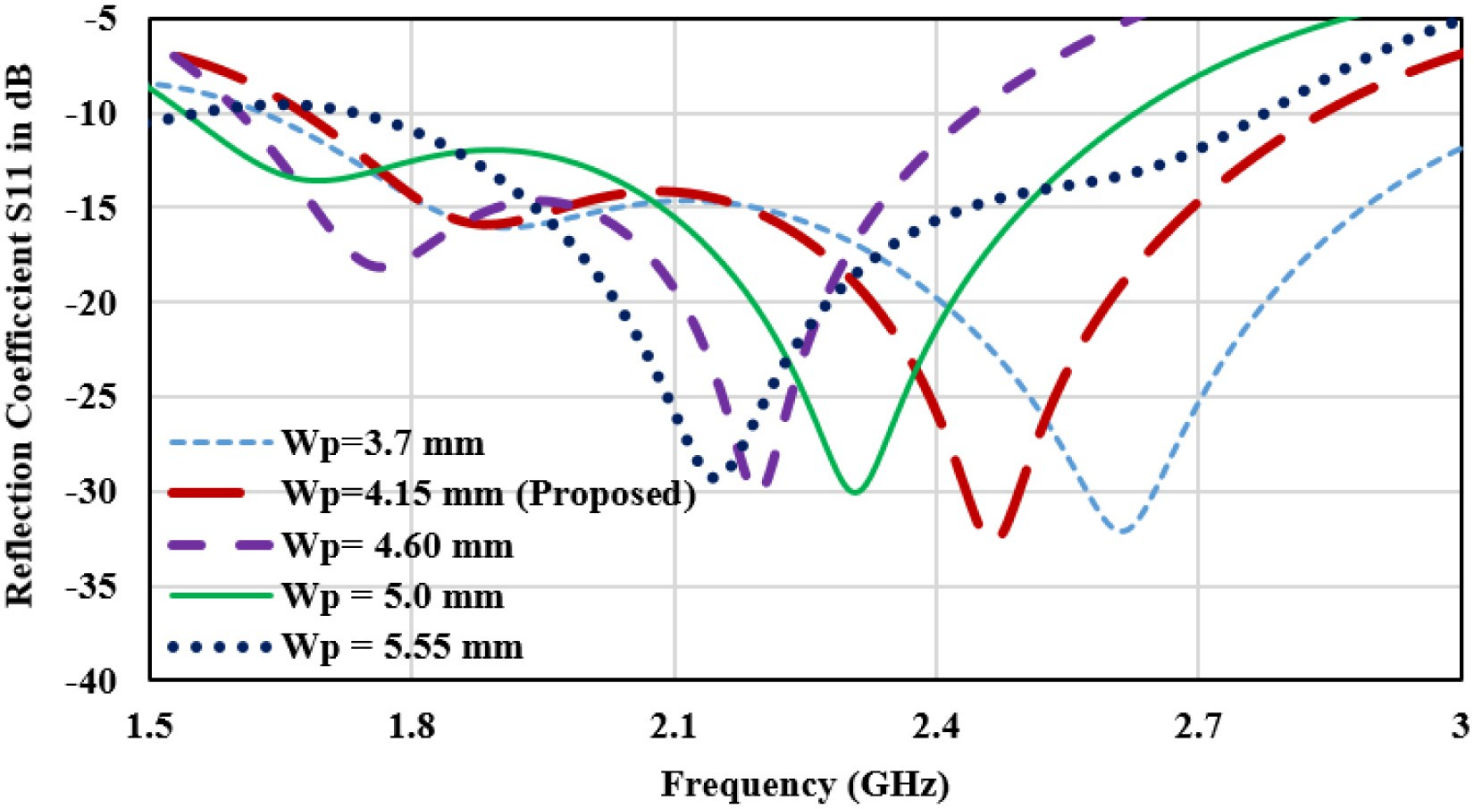
Parametric analysis of the length of the horizontal upper arm of the C-shaped radiator (Wp).

On comparison of the surface current plot of Design2 and Design3, the current moving from the ground of port1 to port2 has altered the path through the decoupling network and reduces the mutual coupling between radiators. In reverse it slightly detunes the central resonance frequency from 2.45 GHz to 2.15 GHz. Therefore, the antenna is further modified for the desired resonance frequency and minimum mutual coupling. To shorten the resonance current path length, the length of the horizontal upper arm of the C-shaped radiator is reduced in Design4. It makes an antenna tune at 2.45 GHz with a bandwidth of 500 MHz from 2.2 GHz to 2.7 GHz with a maximum port isolation of -34 dB. For tuning the antenna at 2.45 GHz, a parametric analysis of the length of the horizontal upper arm of the C-shaped radiator (Wp) is performed. The reflection plot for varying ‘Wp’ is shown in Fig 8, and a significant impact on the frequency and bandwidth can be observed. Frequency reduces with the increasing length and bandwidth is also broadening towards the lower cutoff frequency. The desired frequency is attained at wp = 4.15mm.

Designing implantable antennas requires considering the tissue’s electrical properties. To address this, an equivalent dielectric constant is calculated, which is then used in standard antenna design formulas. The following design equations are considered to design the patch antenna. The width (w) of the patch is calculated by using Eq (1), in which the resonance frequency is (*f*_0_) 2.45 GHz, ‘h’ is the height of the antenna, and (*ε*_*eqv*_) is the equivalent dielectric constant that depends on multiple tissue layers and antenna substrate. Standard antenna theory suggests a half wavelength is needed for resonance. It is difficult to attain resonance with such a compact dimension. The solution lies in altering the patch shape of the antenna. This modification creates a longer electrical path within the limited physical space. In simpler terms, by strategically shaping the antenna, the electrical signal travels a greater distance even though the physical size remains compact. This helps the antenna achieve resonance despite the initial size constraint. The physical length of the antenna at quarter wavelength is calculated according to Eqs (2) and (3).


$$w=\frac{c}{4f_{0}\;\sqrt{\frac{\varepsilon_{eqv}+1}{2}}}$$ (1)

$$\varepsilon_{reff}=\frac{\varepsilon_{eqv}+1}{2}+\frac{\varepsilon_{eqv}-1}{2}{\left[1+12\frac{h}{w}\right]}^{-0.5}$$ (2)

$$l=\;\frac{c}{4\sqrt{\varepsilon_{reff}}}-0.824h\left(\frac{\left(\;\varepsilon_{reff}+0.3\right)\left(\frac{w}{h}+0.264\right)}{\left(\;\varepsilon_{reff}-0.258\right)\left(\frac{w}{h}+0.8\right)}\right)$$ (3)

**Fig 9:**
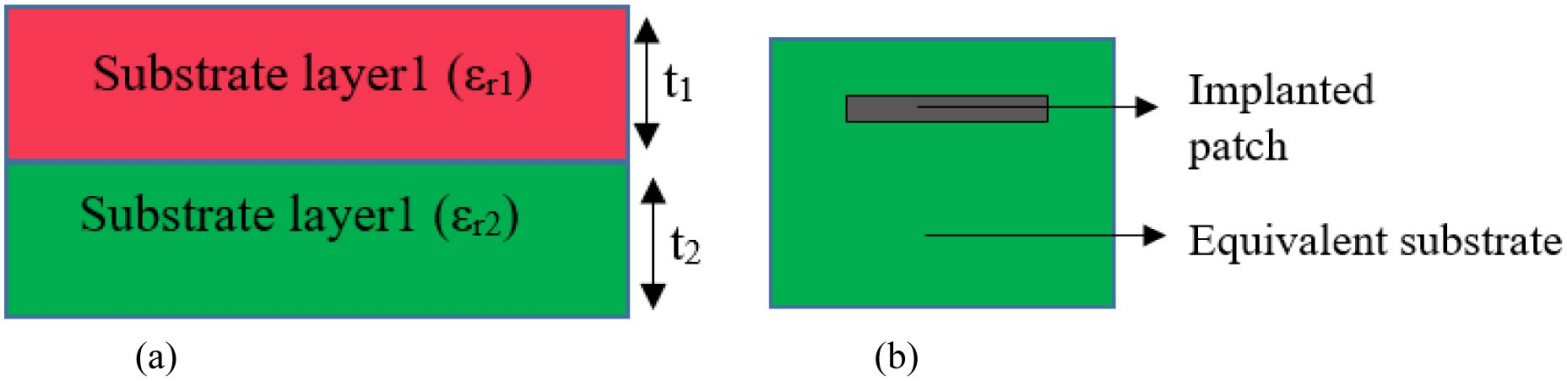
(a) “Krasweski model” and (b) Equivalent single dielectric layer.

Here, *c* is the “speed of light” and *ε*_*reff*_ is the “effective dielectric constant” due to fringing effect of the antenna. To evaluate the fringing effect, the equivalent dialectic constant (*ε*_*eqv*_) needs to be evaluated from complex and lossy multilayered structures with heterogeneous thickness and permittivity. The Krasweski model helps account for the combined influence of multiple tissue layers on the antenna’s performance by providing a single, effective dielectric constant through Eq 4 [48, 49]. Fig 9 shows a method to find the (*ε*_*eqv*_) for 2-dielectric materials. A similar process is followed for other layers.

$$\varepsilon_{eqv}=\;{\left[\sqrt{\varepsilon_{r2}}+vi\left(\sqrt{\varepsilon_{r2}}-\sqrt{\varepsilon_{r1}}\right)\right]}^{2}$$ (4)

Where *ε*_*ri*_ depicts the “relative permittivity” of the i^th^ layer and *vi* gives the surface volume of the i^th^ layer that is evaluated by taking the ratio of the i^th^ layer volume and the total volume.

The effective resonating current length (LR) is the function of the designed antenna geometry, it can be evaluated by addressing the “surface current distribution” represented in Fig 5(h). The current path length from geometric parameters is given in Eq (5).

$$LR=Wp+L2+wp1+L6+Wp2+L4-2\left(L1\right)\;=17.25\;mm$$ (5)

Where, Wn, and Ln are the size of the antenna sides and slots which are listed in Table 2. The resonance frequency is related to radiating path length as given in Eq 6.


$$LR=\;\frac{c}{2f_{r}\sqrt{\varepsilon_{reff}}}$$ (6)

The value of *ε*_*reff*_ is 12.345 evaluated from Eqs (2) and (4). The required half-wave resonance length is 17.70 mm for 2.45 Ghz. It shows that the numerically simulated value (from Eq 6) lies in close approximation with the evaluated current path length from the antenna geometry (Eq 5).

## III. Results and discussions

**Fig 10:**
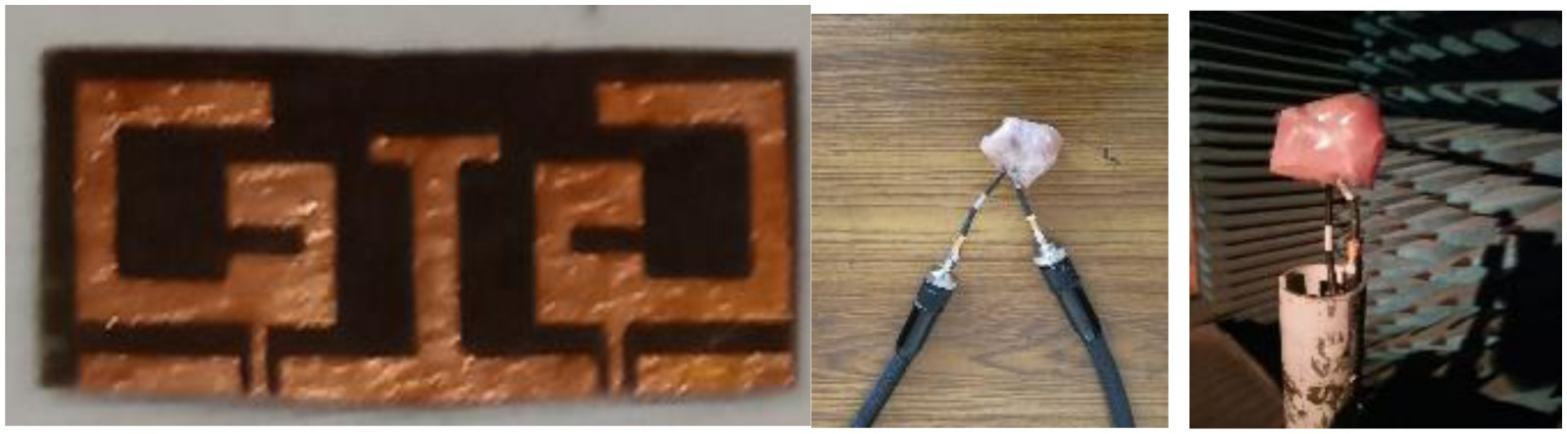
Fabricated prototype and measurement screenshots.

**Fig 11:**
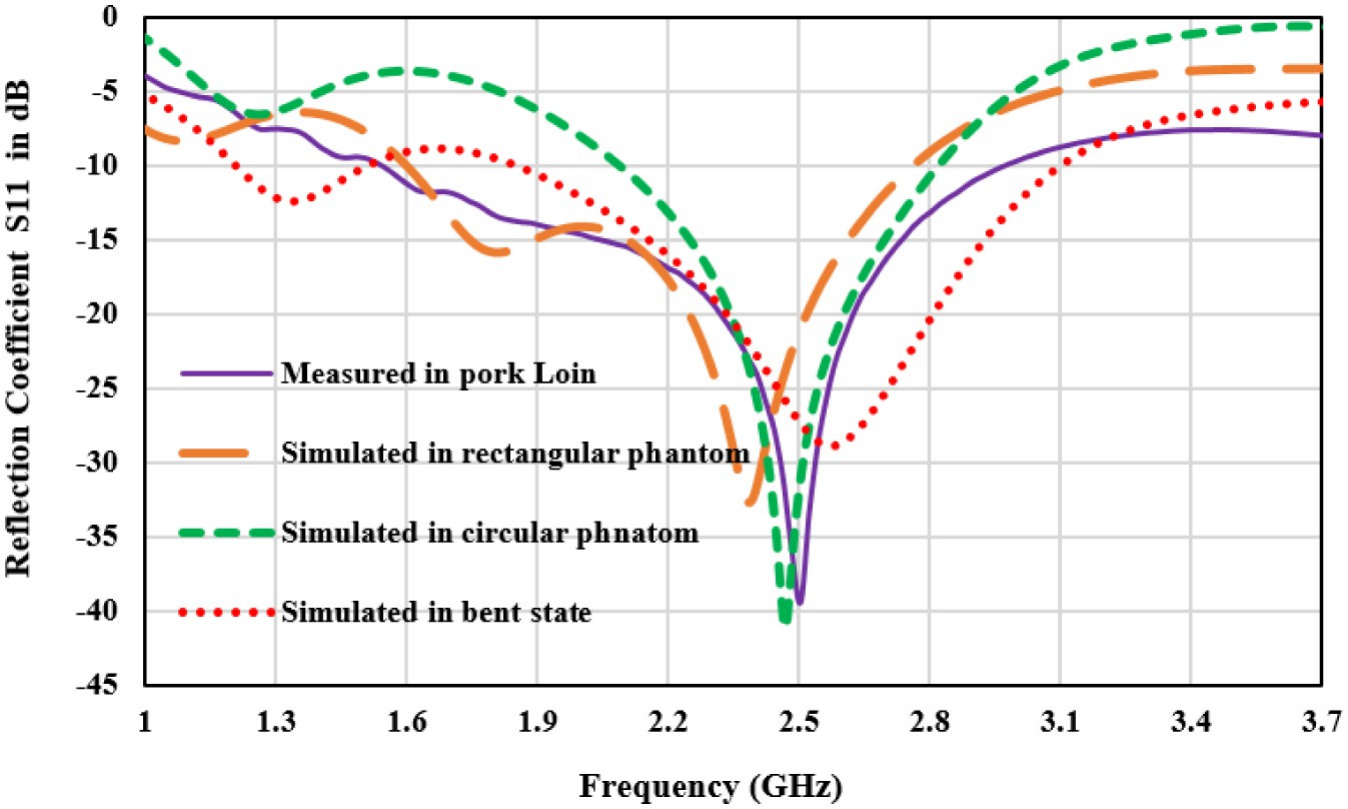
Reflection coefficient plot for measured and different simulation conditions.

**Fig 12:**
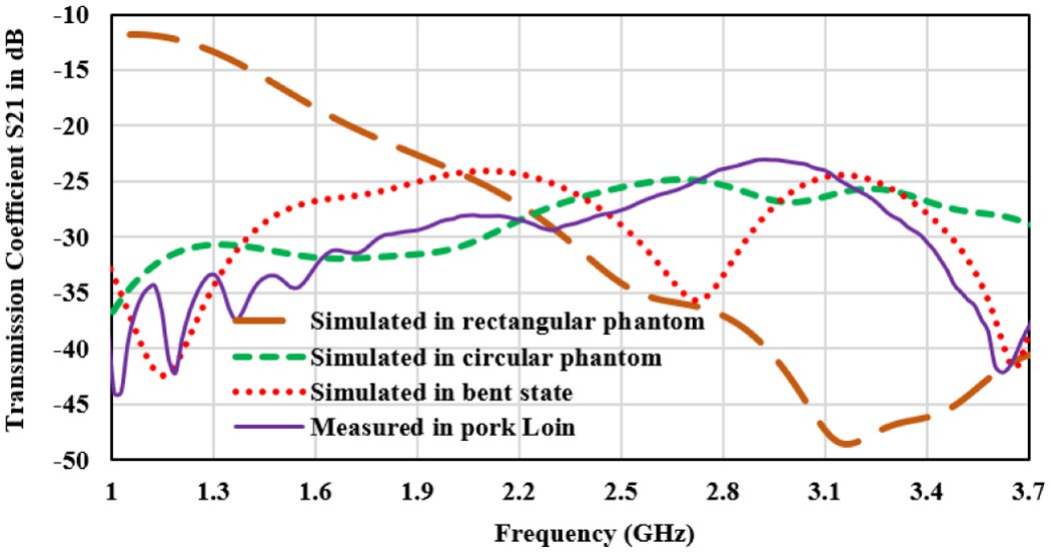
Transmission coefficient plot for measured and different simulation conditions.

To validate the results, antenna is fabricated inserted in the pork tissue for in-body operation. Pictures of the fabricated antenna and experimental setup are presented in Fig 10. In Fig 11, a comparison of the measured “reflection coefficient” and “transmission coefficient” with simulated results is shown. Simulation results for all three operating conditions which are shown in Fig 1 are compared with the measured result. Simulated |S11| plot for the rectangular tissue model shows the 10-dB impedance-bandwidth of 1200 MHz (ranging from 1.6 GHz to 2.8 GHz). The measured graph represents the bandwidth of 1300 MHz (1.6GHz-2.9 GHz). Antenna impedance matching and performance robustness for the real-time in-body operating condition is justified by the |S11| plots in circular heterogeneous phantom and bending the antenna. It can be observed that the antenna operating bandwidth approximately overlapped with the measured results. A slight deviation in operating frequency can be observed towards the lower cut-off. The reflection coefficient value is shifted to -30 dB in the case of the bent antenna as compared to -40 dB in the measured tissue. This is probably due to the variation in internal reflections and diffractions due to curves in the tissue structure. Moreover, Human tissue is a heterogeneous layered structure with different electrical properties. Effective dielectric constant of the antenna is the function of volumetric distribution of the surrounding dielectric materials as given in Eq (4). Surrounding shape of the body tissue of the antenna also varies the scattering and reflection properties of EM waves. It causes the variation in the impedance matching and resonance frequency of antenna for circular and rectangular phantom. However, the antenna has stable and excellent bandwidth to withstand the frequency detuning effect in heterogeneous operating conditions as well as good impedance matching for the ISM band. Port isolation between the two radiators is shown through the transmission coefficient |S21| plot in Fig 12. The |S12 or S21| parameter over the bandwidth is less than -25dB for all thgivene operating conditions. Maximum coupling of -25 dB is obtained in the circular phantom, and for the measured result port coupling of -28 dB is obtained at 2.45 GHz. It depicts that the proposed antenna is well-matched for MIMO operation. |S11| and |S21| are similar to |S22| and |S12| respectively and overlapped. Thus only |S11| and |S21| plots are presented.

**Fig 13:**
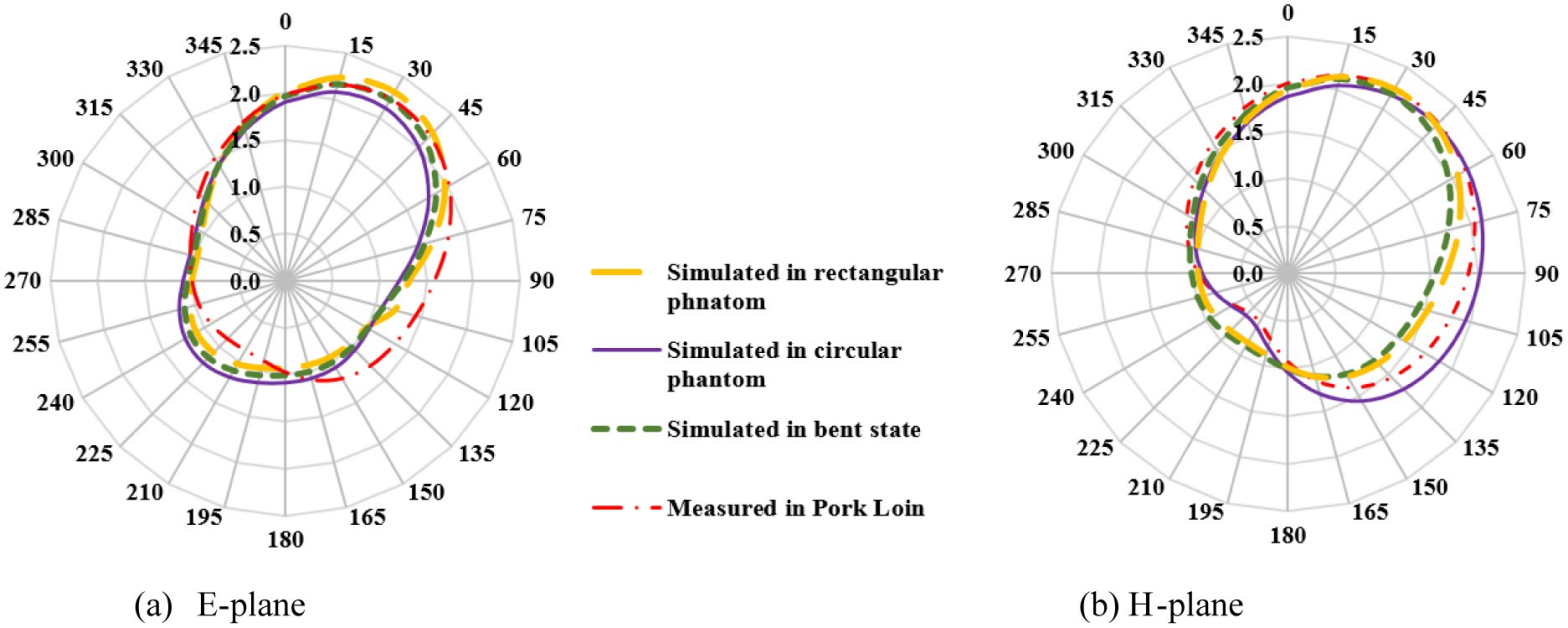
The 2-D radiation pattern of the antenna (a) E-plane (b) H-plane.

**Fig 14:**
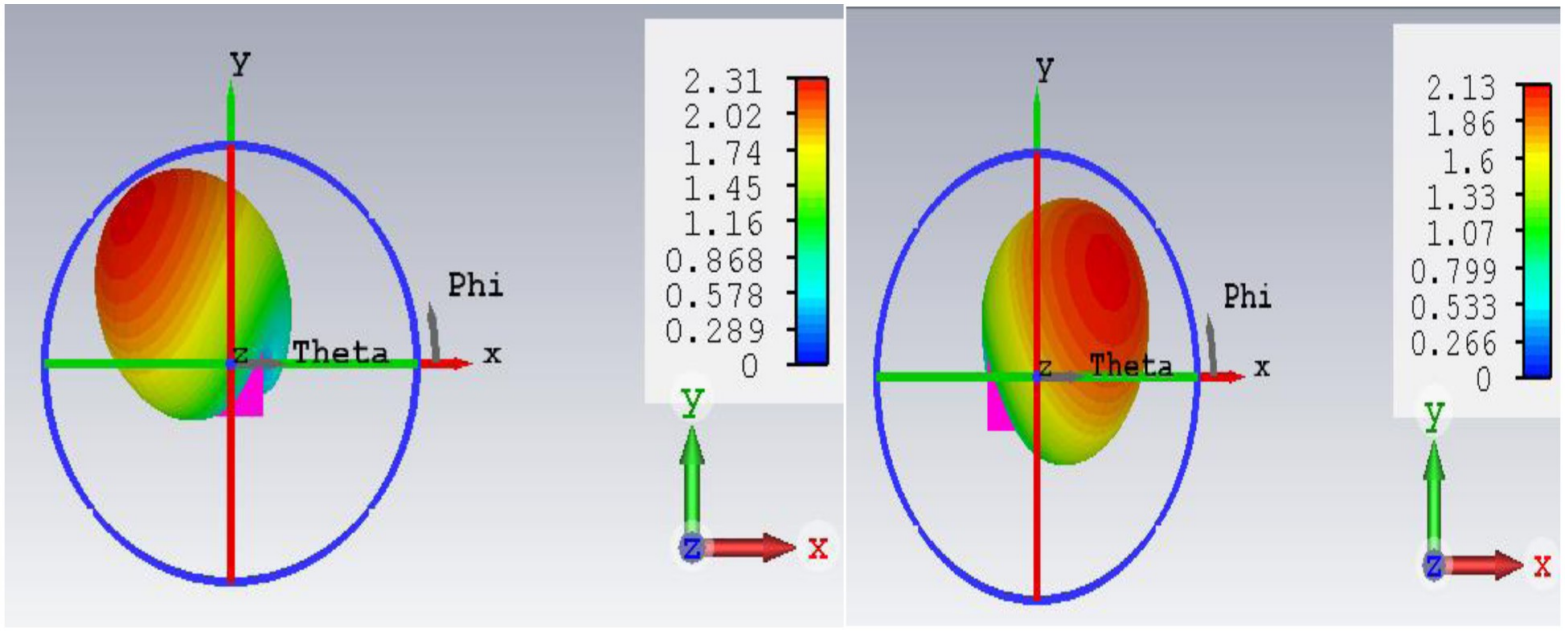
3-D radiation plot of antenna (a) radiator1 (b) radiator2.

**Fig 15:**
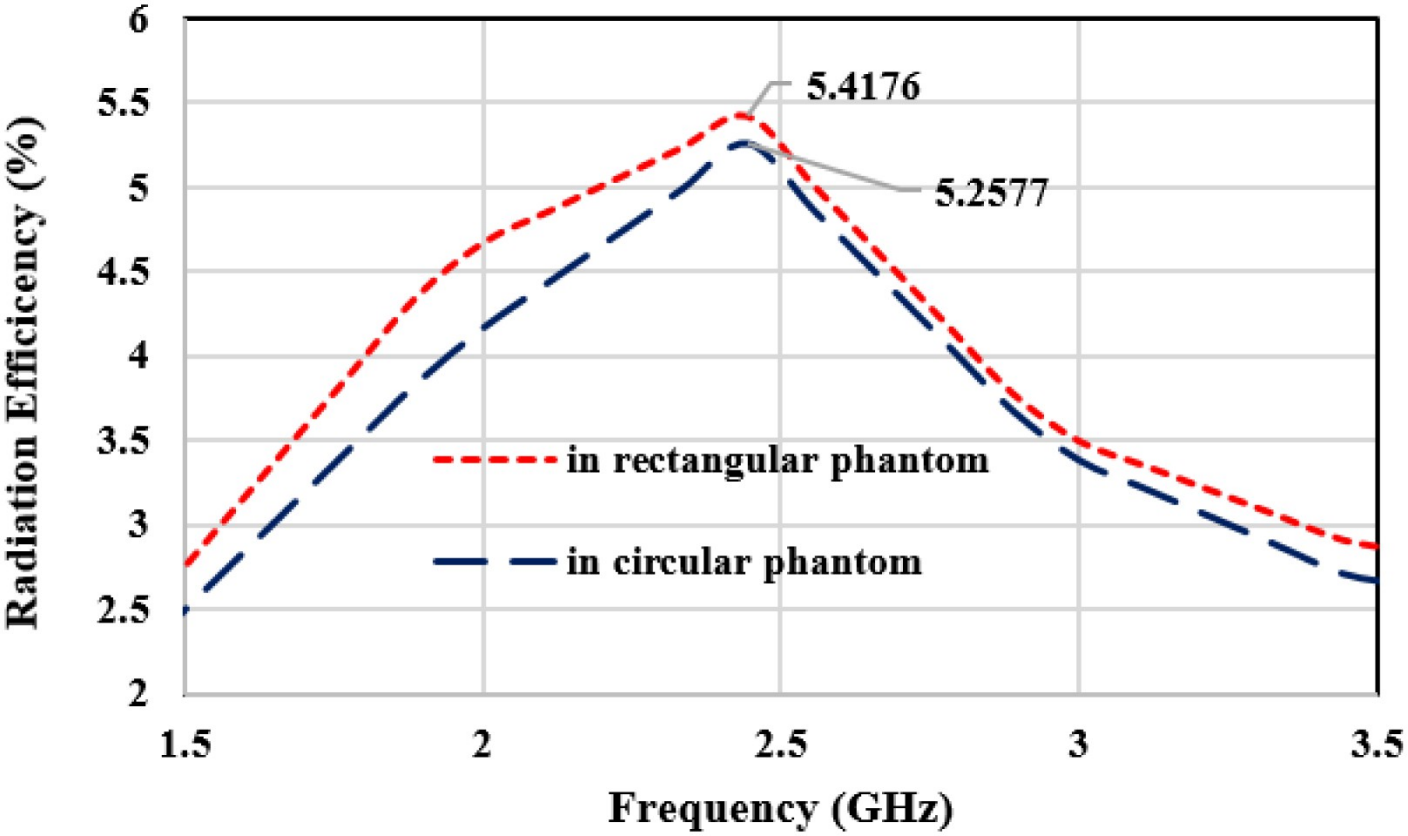
Radiation efficiency of antenna.

2-D radiation characteristics of the antenna for experimental results, simulated rectangular, circular phantoms, and bending conditions are shown in Fig 13. For both the E-plane and H-plane maximum radiations are oriented towards the normal to the surface of the tissue. It is required for the in-body to off-body communication to make a reliable communication link and for reducing the heating effects in tissue due to the absorption of EM waves. The slight difference in the variation of radiation in three operating conditions is because EM waves are the function of thickness and the electric properties of the tissue. It is probable to have some variation in heterogeneous operating conditions. However, the orientation of radiations and the radiated power value are almost similar in all conditions. The measured gain for the antenna is 2.3 dB in both the E-plane and H-plane. Fig 14 depicts the 3-D gain pattern of the antenna for the radiating element1 and element2. It makes sure that the antenna has unidirectional radiation performance with excellent implant gain as compared to the antennas reported in the literature. In addition to this, both radiating elements are radiating in the opposite direction, which reduces the interference between two closely spaced radiators. Radiation efficiency plot of antenna is shown in Fig 15. For the circular phantom efficiency is 5.25% and for the rectangular phantom efficiency is 5.41%.

The antenna is designed for MIMO systems, which use multiple antennas to improve communication performance. To evaluate MIMO performance, two key parameters are analyzed; the first is the envelope-correlation-coefficient (ECC) indicates how similar the radiation patterns of the antennas are. High ECC signifies a strong correlation, which can degrade MIMO performance. For good MIMO operation, ECC should be below 0.05. Another is the Diversity Gain (DG) which relates the improvement in signal with MIMO system [37, 38]. Fairfield-radiation-pattern is used to calculate the ECC value as given in Eq (7).

$$\rho_{e}=\frac{{\left|{∯}_{\Omega }\left[{\vec{E}}_{1}\left(\theta ,\phi \right)\cdot {\vec{E}}_{2}^{*}\left(\theta ,\phi \right)\right]d\Omega \right|}^{2}}{{∯}_{\Omega }{\left|{\vec{E}}_{1}\left(\theta ,\phi \right)\right|}^{2}d_{\Omega }.\;{∯}_{\Omega }{\left|{\vec{E}}_{2}\left(\theta ,\phi \right)\right|}^{2}d\Omega }$$ (7)

Where, ${\vec{E}}_{1}\left(\theta ,\phi \right)$ and ${\vec{E}}_{2}^{*}\left(\theta ,\phi \right)$ are the far-fields of MIMO antenna for the radiating element1 and radiating element2.

Diversity gain shows the execution of diversity-performance of MIMO system and it can be determined through ECC value.


$$DG=10\sqrt{\left(1-\left|\rho_{e}\right|\right)}$$ (8)

**Fig 16:**
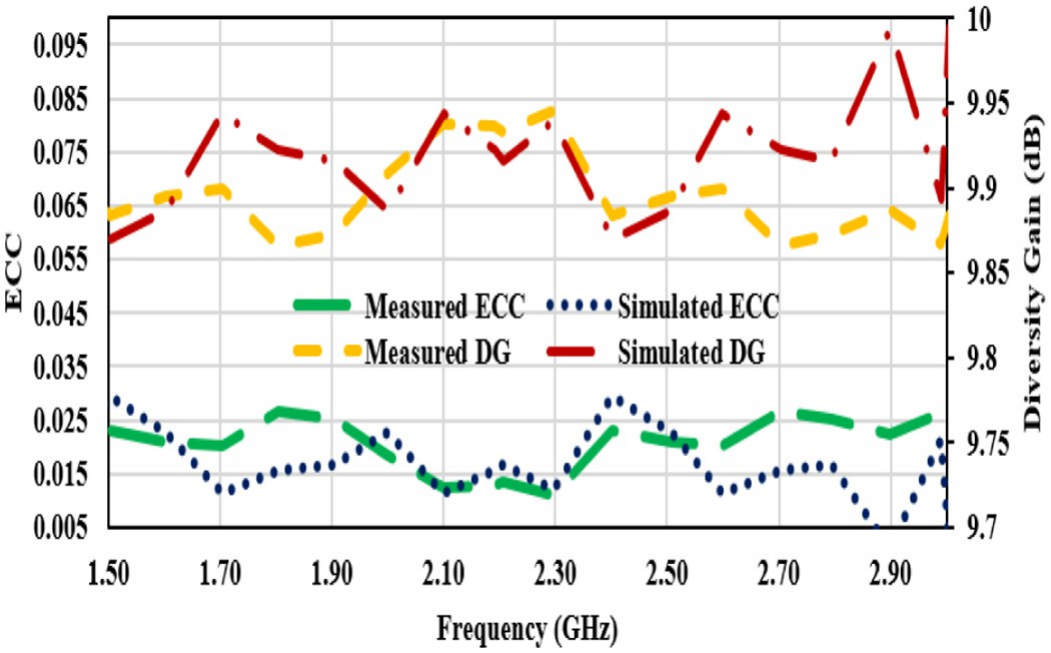
Measured and simulated ECC and diversity gain plots.

Simulated and measured ECC and DG plots for the simulated and measured data are shown in Fig 16. It can be observed that ECC results are well below 0.035 and DG is more than 9.85 dB.

**Fig 17:**
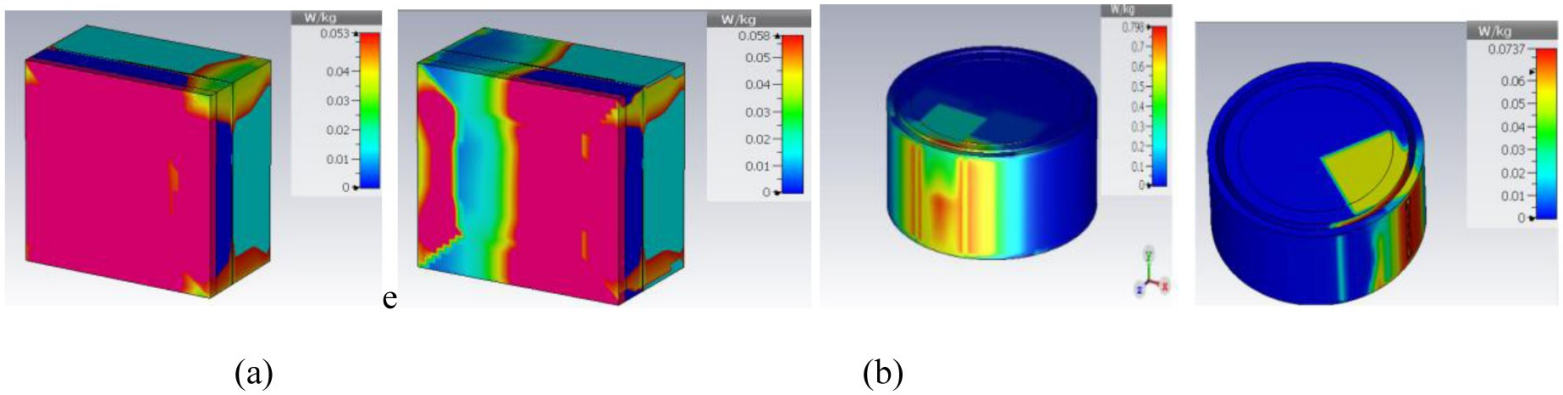
Specific absorption rate plot for the on rectangular phantom (a) port1 (b) port2 and on circular phantom (c) port1 (d) port 2 is excited.

The specific absorption rate is also the most important parameter for on-body applications. To ensure the safety of the user, the SAR value is numerically simulated for port 1 and shown in Fig 17. The proposed structure has SAR values of 0.053 W/Kg and 0.0798 W/Kg for rectangular and circular phantom models respectively. Input power for SAR calculation is considered as 0.1mW for the port1 and port 2 independently. The obtained SAR value is far below the safety limits and makes the antenna safe for body-centric communication. In circular phantom SAR value is higher than the rectangular phantom as, the reflected waves are more concentrated towards the centre of circular shaped tissue due to uniform and symmetrical field distribution. It results in the larger power absorption by circular tissue. Rectangular tissue has sharper edges which leads to more scattering and reflections of EM waves and reduce the power absorption. Thus, SAR value is slightly lower in rectangular phantom.

To analyze the simultaneous effect of SAR by two radiators in MIMO configurations, “SPLSR (Specific Absorption Rate for Peak Distance Location Ratio)” is used. It must be below “0.4 W/Kg/cm” when the space between two radiators is less than 5 cm. SPLSR is given by Eq (9):

$$\text{SPLSR}=\left(\text{SAR}1+\text{SAR}2\right)/\text{D}$$ (9)

Where, SAR1 and SAR2 are the specific absorptions rate of the two radiating elements when powered independently. And D is the center to centre distance between the two radiators. Here the D is 8 mm long X-axis. SPLSR for the rectangular phantom is “0.13875 W/Kg/cm” and for circular phantom is “0.191875 W/Kg/cm” for 0.1mW of input power at each port. The proposed MIMO structure has the SAR value in the safety limits.

Table 3 presents a comparison between the previous works in the field and the proposed work. The present structure has compact and simplest geometry, high port isolation, and excellent radiation characteristics with high gain for in-body conditions.

**Table 3:** Analysis of antenna performance parameter of different implant antenna in literature.

Ref.	[30]	[31]	[33]	[38]	[34]	[41]	This Work
Frequency (MHz)	2450	2450	2450	915,2450	2450	433	2450
Size (mm)	5.36×6.2×0.12	18.5×18.5×1.27	15×3×8	10.8×5.6×0.254	π×(22.9)^2^×0.25	π×(5.65)^2^×0.13	6×16×0.245
Port Isolation (dB)	28	15.99	26.3	24	15	26	29
Gain (dBi)	-20.5	-15.18	-20	-32.15,-22.2	4.2	-30	2.31 dB
ECC	<0.1	0.0025	<0.1	<0.2	<18.8 dB	<0.1	<0.035
Techniques	Meandered Line	EBG	Characteristics Mode Theory and helical radiator	Meandered Line	HIS	Slotted Patch	Slotted patch

## Conclusion

A compact implantable-MIMO antenna with extremely low mutual coupling is designed and analyzed for 2450 MHz ISM band. Two slotted patch resonators are closely positioned with the distance of only 7.7mm and that have the connected ground plane. Size of the MIMO antenna system is scaled by implementing a slotted patch geometry and coplanar-ground-plane. Mutual coupling between the MIMO elements is reduced by using a T-shaped decoupling network with the ground plane. As a result, the antenna has achieved compact dimensions of 24 mm^3^ and high isolation of 29 dB at 2.45GHz. The designed antenna has a 10-dB impedance bandwidth of 1300 MHz (1.6GHz to 2.9GHz) and a gain of 2.3 dBi. Moreover, an SAR value of 0.798 W/Kg. of the proposed antenna with an input power of 0.1mW for 1-g of tissue is achieved. The antenna attains a unidirectional radiation pattern with a wide beam oriented in the opposite direction for the two radiating elements. Based on the realized outcomes, the proposed MIMO antenna is an appropriate choice for implantable medical devices.

## Supporting information

S1 Dataset (XLSX)
